# Korean Nursing Students’ Emotional Response Types to Pandemic: Application of Q-Methodology

**DOI:** 10.3390/healthcare9081080

**Published:** 2021-08-22

**Authors:** Mihyeon Seong

**Affiliations:** Department of Nursing, Changshin University, Changwon 51352, Korea; mihyeon0624@cs.ac.kr; Tel.: +82-55-250-3165

**Keywords:** Q-methodology, nursing students, pandemic, emotional responses

## Abstract

The aim of this study is to investigate the emotional responses of nursing students to the COVID-19 pandemic and the characteristics of these responses using the Q-methodology. The focus of the Q-methodology is to discover research participants’ subjective viewpoints. In May 2021, 50 Korean nursing students from first year to fourth year were selected to participate in the study, and data were collected by asking them to classify 37 selected Q-statements in a normal distribution on a 9-point scale. In the final analysis, a P-sample of 38 nursing students was used. The emotional responses of the Korean nursing students were categorized into four types: self-protection (Type 1), pessimism about the current situation (Type 2), realistic optimism (Type 3), and developmental-seeking (Type 4). The four factors accounted for 39% of the total variance. The individual explanatory powers of the four factors were 8%, 12%, 13%, and 6%, respectively. Thus, the study explored the subjectivity of emotional responses of Korean nursing students to the pandemic. The study recommends considering the results for intervention programs that are designed to prepare nursing students for future health crises and pandemics.

## 1. Introduction

### The Rationale for This Research

In December 2019, a novel coronavirus (SARS-CoV-2) caused an outbreak of coronavirus disease (COVID-19) in Wuhan, China, which then spread worldwide in three months, leading the World Health Organization (WHO) to declare it a global pandemic [1]. The COVID-19 pandemic inflicted tremendous burdens of morbidity and mortality on the world, while critically disrupting economies and societies globally [2]. Several factors such as uncertain prognoses, an unfamiliar environment that infringes on personal freedom, and a lack of resources for testing and treating patients placed a great deal of stress on people. All of this also contributed to an increase in emotional distress and psychiatric disorders, especially among healthcare providers and others responding to cases of infection [3]. For students, the COVID-19 pandemic affected their academic performance and many were unable to complete their curriculum requirements and assessments proficiently [4]. Nursing students, in particular, are more likely to experience emotional difficulties due to their rapidly changing clinical field. Their extensive curriculum has increased their workload compared to other departments and this has made preparing for the national nursing licensure examination tougher. [5].

In addition, the medical community is working with the government to set up screening clinics to prevent the spread of COVID-19. Healthcare providers sent to work in these clinics are at risk of infection [6]. Owing to the concerns that nursing students who receive practical training under such circumstances may also be exposed to the risk of infection [7], hospitals are compelled to limit practical training by substituting it with in-school training [8]. Clinical training, which includes practical training, constitutes a large part of nursing education, and its suspension or inadequacy due to COVID-19 may harm nursing students by provoking feelings of uncertainty, anxiety, and stress, instigating their withdrawal from the nursing program [9,10]. It has been reported that nursing students in particular perceive themselves to be at a higher risk of infection during an epidemic [10] and consequently have a lower willingness to care for patients when they experience higher levels of stress [11]. In addition, although the employment rate of nursing students is higher than that of students from other departments, nurses have a high early turnover rate, and the number of students who are unemployed after graduation is increasing [11]. As a result, the emotional responses of nursing students (who are future healthcare providers) to the pandemic must be examined. There are several studies on nursing students in the COVID-19 situation, but this knowledge is still insufficient [7]. However, there are no studies that present the students’ viewpoints about the pandemic or their responses to the COVID-19 situation.

In South Korea, people in their 20s accounted for 21% of confirmed COVID-19 cases, while people in their 50s and 60s accounted for 18.3% and 15.2%, respectively; thus, people in their 20s accounted for the largest percentage of confirmed cases [12]. As most nursing students in South Korea are in their 20s, it is crucial to examine their emotional responses to the pandemic and develop effective interventions that reflect the characteristics of the younger generation. Examining the emotional responses of nursing students to the pandemic can also provide valuable insights into managing and controlling the spread of infectious diseases [1]. Moreover, when people experience adversities like the COVID-19 pandemic, some find and develop new strengths, while others may develop psychiatric problems such as post-traumatic stress disorder (PTSD) [3]. Exploring and understanding the cultural and social contexts of nursing students can help in designing appropriate emotional support that can maximize their strengths and minimize their weaknesses. Consequently, this can serve as a basis for devising response strategies to cope with existing infectious diseases and the ongoing global threat of new infectious diseases that nursing students may encounter in the future. For this purpose, the current study uses the Q-methodology, which was developed to systemically study the viewpoints of people involved in a phenomenon [13].

The Q-methodology compensates for objective bias in qualitative research. It provides a subjective interpretation, by allowing the respondents to compare the statements, determine the order, and model them to express their own subjectivity. This allows subjective viewpoints to be measured in an objective way through integrative theory and a factor analysis [14,15]. Q-methodology is used to find correlations between people, identify and categorize the differences in their subjective perceptions, and classify them into groups according to the similarities in their thoughts, attitudes, and values about a subject or phenomenon [16]. This method compensates for the weakness of one method with the strength of the other method [17]. Therefore, this study attempts to categorize the characteristics of the emotional responses of nursing students to the pandemic by applying the Q-methodology.

## 2. Materials and Methods

### 2.1. Research Design

The present study is an exploratory study that applies the Q-methodology to conduct an in-depth systematic and scientific investigation of the emotional responses of nursing students to the COVID-19 pandemic. The Q-methodology uses a statistical technique based on correlation and a factor analysis to test a group of variables on multiple people at a certain time. The scores are used to calculate the correlation coefficient between people. This is different from quantitative statistical methods in which multiple people are tested on multiple variables and then the correlation coefficient between two variables is calculated [15]. The factor analysis in quantitative statistical techniques seeks differences between individuals, whereas Q-methodology seeks intra-individual differences. In the Q -methodology, an individual assigns a score to an item that has a strong psychological meaning to her/him and measures his or her subjective opinion, thereby making it possible to explore the inclinations and viewpoints of individuals or small groups [15,18].

### 2.2. Research Procedure

#### 2.2.1. Q-Population Construction and Q-Sample Selection

The Q-sample for this study was constructed using the literature review and interviews. In general, about 10 interviews are sufficient to form a Q-population. If the statements start duplicating, further interviews become meaningless, and the integration is considered to be complete [14]. The semi-structured interviews included questions such as, “How do you feel about the pandemic situation?”, “How do you think the pandemic situation will develop in the future?”, “How would you feel if you went to the clinic from the current situation?”, “What is the most important thing in a pandemic situation?”, etc., were used to understand the participant’s viewpoint. A total of 267 Q-populations were derived from 108 statements extracted through semi-structured interviews and 159 statements were derived from the literature review. The initial arrangement of items from literature was self-referential and later were arranged and classified by the participants. Questions with overlapping meanings were deleted, and statements with similar meanings were arranged and categorized. The final Q-sample consisted of 37 statements.

#### 2.2.2. P-Sample

Unlike existing deductive methodology, the Q-methodology does not aim at generalization, but rather for hypothesis inference [14]. It seeks differences within individuals and is a useful research method for a small and inmate sample analysis. However, this methodology is not limited by the P-sample. On the contrary, when the P-sample becomes large, a number of people concentrate on one factor, which causes a statistical problem and characteristics cannot be clearly identified [14]. Usually, around 50 people are used [14,15]. The participants, the P-sample, for this study were 1st to 4th year nursing students attending University C and University J in Korea. In addition, a total of 50 people, including those who had already participated in the interview, were conveniently sampled to form the Q-population.

#### 2.2.3. Q-Classification Process and Method

**Figure 1:**
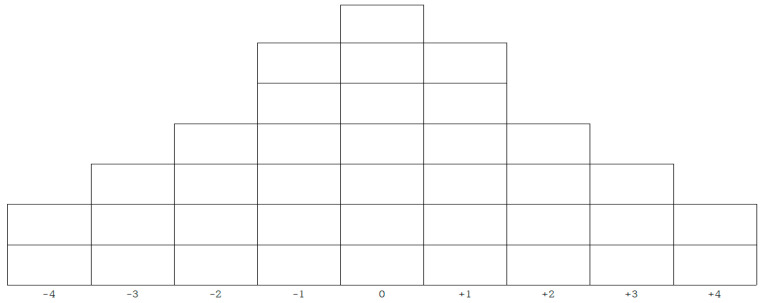
Card arrangement distribution table.

The process of Q-classification was carried out by trained research assistants with nursing licenses. The process lasted from 1 May 2021 to 20 May 2021. It included investigating general characteristics and conducting interviews, which took approximately an hour for each participant. To perform Q-classification, each statement of the finalized Q-sample was written on a paper card (Q-statement card), and the cards were numbered from 1 to 37. The study participants were invited to read and sort each of the Q-statements into the following three groups: agree (+), neutral (0), and disagree (−). After classifying the Q-statements, the participants were asked to read and rank the statements that had been sorted into the “agree” group on a scale of most agree (+4) to neutral (0). Similarly, the participants were asked to rank the Q-statements that had been sorted into the “disagree” group on a scale of disagree (−4) to neutral (0) and then arrange them according to the Q-sort table depicted in Figure 1. After the classification process, valuable information for the Q-factor analysis was obtained by asking the participants questions about the reasons and feelings behind their classification. Furthermore, the interviews were recorded on a digital recorder, with the participants’ consent, and the recordings were used for interpreting the results. While the participants arranged the cards for Q-classification, the researcher sat near the participant to instruct the classification method and collected data on general characteristics such as age, gender, and personality. This is based on a previous study that showed that gender and personality influenced emotional response patterns [19]. It took approximately 30 min to one hour to complete the Q-classification process. This also included answering the questionnaire and an interview where participants shared their reasons for Q-classification.

### 2.3. Ethical Considerations

This study was conducted after obtaining approval from the Institutional Review Board of C University (IRB No. CSIRB-R2021016). In order to avoid ethical issues, a trained research assistant explained the purpose of the study, research method, interview, etc. Written consent before the interview and during the Q-sample classification was obtained. In addition, the study participants were reassured that their anonymity would be guaranteed and that the collected data would not be used for any purpose other than for the study. They were also informed of their right to withdraw from the study at any time. Moreover, all the participants received a small gift, as an incentive to increase the survey response. The collected data were sealed and stored on a password-protected hard drive that could only be accessed by the principal investigator. It was decided that this data would be retained for three years, from the time that the data were collected until the publication of the thesis. The data will be destroyed after the publication.

### 2.4. Method of Data Analysis

The study used PQ Method software to perform a principal component analysis. The PQ Method program is a software package dedicated to Q-methodology. It creates a factor sequence table and facilitates a factor analysis that is unique for Q-methodology [15]. The factor analysis is a method of classifying variables in general. In order to classify subjects, a factor analysis is performed, and in this study the commonly used main factor analysis method was used. The 37 Q-statements and their scores were entered into the PQ Method software. The 37 Q-statements were assigned a score (according to the level of agreement or disagreement of each subject in the P-sample) that ranged from −4 points for statements that subjects disagreed with the most and 0 points for neutral statements to 4 points for statements that subjects agreed with the most. This study determined the optimal number of factors by selecting factors with eigenvalues greater than 1.0. Next, to analyze the types of emotional responses that nursing students in South Korea have to the pandemic, this study analyzed the z-scores of the Q-items. The factor weight for each type of emotional response, and the general characteristics of the P-sample were also studied. At this time, in order to prevent type I error, Ricci’s research procedure [20] was referred to, and semantic statements and subject extraction tables were prepared. This also minimized distortions, that could occur because of the researcher’s own bias, through re-evaluation and through triangulation. Various experts participated in the review process to increase the validity and reliability of the study. After synthesizing all the contents, the researcher named the final type.

## 3. Results

### 3.1. Formation of Q-Types

The results of the factor analysis that focused on the P-sample categorized South Korean nursing students’ emotional responses to the pandemic into four factors. The responses were classified into four factors using PQ Method software, and the correlation and explanatory power of the factors were considered. The four factors accounted for 39% of the total variance, with the explanatory power of each factor being, 8% for Factor 1, 12% for Factor 2, 13% for Factor 3, and 6% for Factor 4 (Table 1). Thus, Factor 1, Factor 2, Factor 3, and Factor 4 of the factor analysis were categorized as Type 1, Type 2, Type 3, and Type 4, respectively. After synthesizing all the contents, the researcher named these types. The four types of emotional responses observed among the Korean nursing students were self-protection (Type 1), pessimism about the current situation (Type 2), realistic optimism (Type 3), and developmental-seeking (Type 4).

Of the questionnaires completed by the 50 participants in the P-sample, three copies with insincere responses were excluded, and the remaining 47 were used in the analysis. In addition, nine of the participants (P-2, 7, 12, 16, 20, 27, 29, 35, 41) were not grouped into one of the four types because the differences in factor weights were not significant. Among the participants, 10 were male and 28 were female. While the subject was performing Q-classification, the researcher sat near the subject to instruct on the classification method and collected data on general characteristics such as age, gender, and personality. This is based on a previous study that showed differences in emotional response patterns by gender and personality [19]. All of the 38 participants were classified using the four types of emotional responses. Type 1, Type 2, Type 3, and Type 4 had 6, 15, 12, and 5 participants each. Among the P-samples for each type, a person with a high factor weight contributed to the classification of the characteristics as well (Table 2).

### 3.2. Characteristics by Type of Emotional Response

#### 3.2.1. Type 1: Self-Protection

A total of six nursing students belonged to the Type 1 group, which consisted of one male and five female students aged between 28–30 years (Table 2). Concerning the academic year, one participant was a first-year nursing student, three were second-year students, and two were third-year students. As for the personality type, one participant was extroverted, while the other five participants were of a mixed personality. Regarding the motives for choosing the nursing department, one participant’s decision was based on the recommendation of others, one participant’s decision was based on their college admission scores, two participants’ decisions were voluntary based on their initiative, and two participants’ decisions were based on ease of employment. Of the participants, two had a good degree of satisfaction with regard to their major, and four had an average degree of satisfaction (Table 2).

The Type 1 group strongly agreed with the following Q-items: “Q3. I hope the pandemic will end before I become a nurse (Z = 1.75)”, “Q2. I am afraid that I may spread the infection to others and be stigmatized as a spreader of an infectious disease (Z = 1.56)”, and “Q26. I feel proud to be a nursing student when I see hard-working nurses (Z = 1.53)” (Table 3). While Type 1 participants were concerned about the pandemic, they often exhibited a defensive personality when trying to gather more information about the pandemic. This group was more conscious about the perceptions of other people compared to the other types. This can be confirmed from the following Q-items that Type 1 agreed with more strongly (Z diff ≥ 1.00) than the other types: “Q2. I am afraid that I may spread the infection to others and be stigmatized as a spreader of an infectious disease (Z diff = 1.36)”, and “Q32. As a nursing student, I feel that I should approach information objectively (Z diff = 1.09)” (Table 4). On the other hand, the Type 1 group strongly disagreed with the following Q-items: “Q10. I experienced physical symptoms due to the pandemic, such as loss of appetite and indigestion (Z = −2.36)”, and “Q19. My expectations of college life were shattered (Z = −1.33)” (Table 3). When compared to the other types, Type 1 demonstrated a lower level of agreement (Z diff ≤ 1.00) with “Q20. I am confused about whether I am doing well (Z diff = 1.20)” (Table 4).

Participant number 4, who had the highest factor weight (0.60) in the Type 1 group, agreed the most with the following Q-item: “Q30. It allowed me to think about the nurses’ sense of duty”. They stated that their reason for selecting this item was: “When I saw nurses working actively amid the pandemic, I realized that making unconditional sacrifices is not equivalent to having a sense of duty; however, I think it would be less exhausting if I made reasonable compromises with reality and put myself first while putting patients second”. Moreover, Participant number 4 stated that they disagreed most with the Q-item “Q4. Everyday places have changed into places of fear”, because, in their words, “the places where you spend your daily life, such as your home and school are the best managed in terms of infection control; they are the places where I feel at ease even in a pandemic”.

This study collectively examined the subjective reasons for the selection of Q-statements by the P-sample participants, and the distribution of statements that the subjects agreed or disagreed with for this type of emotional response. As a result, it was determined that the Type 1 emotional response of nursing students to the pandemic, namely self-protection, was characteristically different from the other three types of responses because it was characterized by the following: “I need to study to protect myself, I am afraid of the stigma that follows when a person is unable to protect themselves and becomes infected with an infectious disease”. The Type 1 emotional response was named the “self-protection type”, as it demonstrated a greater tendency to respond defensively than the other types, i.e., by being conscious of the perceptions of people during a pandemic.

#### 3.2.2. Type 2: Pessimism about the Current Situation

A total of 15 nursing students belonged to the Type 2 group, which consisted of two male and thirteen female students aged 18–25 years. Three participants were first-year nursing students, eight were second-year students, and four were third-year students. As for the personality type, three participants were extroverts, four participants were introverts, and eight participants had a mixed personality. Regarding their motive for choosing nursing, one participant’s decision was based on the recommendation of others, nine participants’ decisions were voluntary based on their initiative, and five participants’ decisions were based on the ease of employment. Nine participants were well satisfied with their major, and six had an average degree of satisfaction (Table 2).

The Type 2 group strongly agreed with the following Q-items: “Q3. I hope the pandemic will end before I become a nurse (Z = 1.85)”, “Q17. I am worried about being unprepared as a nursing student (Z = 1.56)”, and “Q7. I am depressed that my activities are being restricted due to the pandemic (Z = 1.44)” (Table 3). In addition, the Type 2 group exhibited feelings of concern and depression, as well as frustration and confusion regarding restrictions on their freedom. This can be confirmed from the following Q-items that Type 2 agreed with more strongly (Z diff ≥ 1.00) than the other types: “Q17. I am worried about being unprepared as a nursing student (Z diff = 1.03)”, and “Q7. I am depressed that my activities are being restricted due to the pandemic (Z diff = 1.44)” (Table 4). On the other hand, the Type 2 group strongly disagreed with the following Q-items: “Q10. I experienced physical symptoms due to the pandemic, such as loss of appetite and indigestion (Z = −2.32)” and “Q16. I regret enrolling in the nursing program (Z = −1.91)” (Table 3). When compared to the other types, Type 2 demonstrated a lower level of agreement (Z diff ≤ 1.00) with the following Q-items: “Q35. I think the current pandemic is a learning ground that will help us cope with future crises (Z diff = 1.37)”, and “Q29. I feel a sense of accomplishment in completing this stage of education despite difficult circumstances” (Z diff = 1.07) (Table 4).

Participant number 1, who had the highest factor weight (0.68) in the Type 2 group, agreed most with the following Q-item: “Q7. I am depressed that my activities are being restricted due to the pandemic”. They stated that their reason for selecting this item was: “In the school campus, I am restricted from accessing certain areas by professors; thus, I have to find my way every time. I still feel like a minor despite being a college student”. In addition, Participant number 1 stated that they disagreed most with the Q-item “Q10. I experienced physical symptoms due to the pandemic, such as loss of appetite and indigestion”, because of the following reason: “As no one around me was infected, it did not affect me much. I am eating and sleeping well despite the pandemic”.

The collective examinations of the subjective reasons determined that the emotional response of the Type 2 nursing students to the pandemic was pessimism about the current situation. This was different from the other three types of responses because it was characterized by the following: “Nothing has changed as a result of the pandemic; it seems like someone else’s business. However, I am unable to do the things that I want to do, and it seems as though the quality of education has decreased due to virtual classes”. When compared to the other types of responses, this type demonstrated a bystander attitude that resembled a third-party standpoint due to the feeling that the pandemic was relevant to others or the outside world, but irrelevant to oneself. Moreover, it also demonstrated the participants’ tendencies to be apathetic as the pandemic continues to spread. In addition, participants exhibiting this type of emotional response attributed their lack of preparation as nursing students to the educational environment, which had undergone several changes due to the pandemic, and they also expressed negative emotions toward the patients of confirmed cases. Therefore, the Type 2 emotional response was named “pessimism about the current situation”.

#### 3.2.3. Type 3: Realistic Optimism

A total of 12 nursing students belonged to the Type 3 group, which consisted of five male and seven female students aged 18–30 years. Concerning the academic year, three participants were first-year nursing students, five were second-year students, and four were third-year students. As for the personality type, five participants were extroverts, four participants were introverts, and three participants were of a mixed personality. Regarding their motives for choosing the nursing department, one participant’s decision was based on the recommendation of others, eight participants’ decisions were voluntary based on their initiative, and three participants’ decisions were based on ease of employment. Of the participants, five had a good degree of satisfaction with regard to their major, and seven had an average degree of satisfaction (Table 2).

The Type 3 group strongly agreed with the following Q-items: “Q33. I look forward to becoming a nurse and participating in social activities as a professional (Z = 2.48)”, “Q26. I feel proud to be a nursing student when I see hard-working nurses (Z = 1.40)”, and “Q34. It feels nice to have unexpected spare time (Z = 1.24)” (Table 3). The Type 3 group exhibited a positive response to the spare time they obtained as their social activities had been limited due to the pandemic. This can be confirmed by the following Q-items that Type 3 agreed with more strongly (Z diff ≥ 1.00) than the other types: “Q33. I look forward to becoming a nurse and participating in social activities as a professional (Z diff = 1.63)”, and “Q34. It feels nice to have unexpected spare time (Z diff = 1.84)” (Table 4). On the contrary, the Type 3 group strongly disagreed with the following Q-items: “Q16. I regret enrolling in the nursing program (Z = −1.91)”, and “Q10. I experienced physical symptoms due to the pandemic, such as loss of appetite and indigestion (Z = −1.73)” (Table 3). When compared to the other types, Type 3 demonstrated a lower level of agreement (Z diff ≤ 1.00) with the following Q-item: “Q2. I am afraid that I may spread the infection to others and be stigmatized as a spreader of an infectious disease (Z diff = 1.22)” (Table 4).

Participant number 8, who had the highest factor weight (0.65) in the Type 3 group, agreed most with the following Q-item: “Q36. It was nice to spend more time with my family due to the pandemic”. They stated that their reason for selecting this item was as follows: “Before the pandemic, I did not find time to even talk to or eat with my family because of my busy schedule; however, as I was at home during the pandemic, I spent time with my family and learned new things about them, which was nice”. In addition, Participant number 8 stated that they disagreed most with the Q-item “Q1. I am suspicious that the people I come into contact with may be infected”, because of the following reason: “Even though the number of confirmed cases is rising, I think it would be insensitive, in terms of interpersonal relationships, to think of the people around you as potentially infected and to act carefully around them”.

This study collectively examined the subjective reasons for the selection of Q-statements by the P-sample participants, as well as the distribution of statements that the participants agreed or disagreed with for this type of emotional response. As a result, it was determined that the Type 3 emotional response of nursing students to the pandemic, namely realistic optimism, was characteristically different from the other three types of responses because it was characterized by the following: “I try to be productive in the spare time that I got due to restrictions that were implemented, such as the practice of social distancing and the ban on group gatherings”. The Type 3 emotional response was named “realistic optimism”, as it demonstrated a more positive attitude toward the pandemic, as well as a more receptive attitude in terms of adjusting to the changing educational environment compared to the other types.

#### 3.2.4. Type 4: Developmental-Seeking

A total of five nursing students belonged to the Type 4 group, which consisted of two male and three female students aged 19–23 years. Concerning the academic year, one participant was a first-year nursing student, two were second-year students, one was a third-year student, and one was a fourth-year student. As for the personality type, two participants were introverts, and three participants were of a mixed personality. Regarding the motives for choosing the nursing department, all five participants answered that they had made the decision voluntarily based on their initiative (Table 2).

The Type 4 group strongly agreed with the following Q-items: “Q23. We will be able to experience personal growth by overcoming the pandemic (Z = 1.42)”, “Q30. It allowed me to think about the nurses’ sense of duty (Z = 1.38)”, and “Q37. I think my competence as a professional nurse will increase as a result of experiencing new methods of teaching and learning (Z = 1.26)” (Table 3). The Type 4 participants thought that overcoming a crisis in the pandemic could help them experience growth as a nurse. This can be confirmed by the following Q-item that Type 4 agreed with more strongly (Z diff ≥ 1.00) than the other types: “Q37. I think my competence as a professional nurse will increase as a result of experiencing new methods of teaching and learning (Z diff = 1.28)” (Table 4). On the contrary, the Type 4 group strongly disagreed with the following Q-items: “Q34. It feels nice to have unexpected spare time (Z = −2.28)”, and “Q16. I regret enrolling in the nursing program (Z = −1.95)” (Table 3). Moreover, when compared to the other types, Type 4 demonstrated a lower level of agreement (Z diff ≤ 1.00) the following Q-item: “Q34. It feels nice to have unexpected spare time (Z diff = 1.68)” (Table 4).

Participant number 35, who had the highest factor weight (0.60) in the Type 4 group, agreed most with the following Q-item: “Q14. As a nursing student, it infuriates me to see people behaving irresponsibly”. They stated that their reason for selecting this item was: “It infuriates me to see people behaving irresponsibly while healthcare workers are working hard on the front lines to help infected patients”. The Q-item that they disagreed with most was: “Q6. I am concerned that the quality of nursing education will decrease due to the pandemic”. Their reason for selecting this item was: “On the contrary, I think the quality of education is improving as the school is providing us with in-depth knowledge about the infection due to the gravity of the situation. Therefore, I think we would be able to demonstrate our professional competence on the front lines as soon as we obtain our nursing license”.

This study collectively examined the subjective reasons for the selection of Q-statements by the P-sample participants, as well as the distribution of statements that the participants agreed or disagreed with for this type of emotional response. As a result, it was determined that the Type 4 emotional response of nursing students to the pandemic, namely developmental-seeking, was characteristically different from the other three types of responses because it was characterized by the following: “We should use the present day as a lesson for the future and strive for progress”. The Type 4 emotional response was named “developmental-seeking”, as it demonstrated the willingness to strive for progress despite the pandemic and prepare for the future with a progressive mind.

In conclusion, it was found that the four types of emotional responses exhibited both independent and mutually coexistent characteristics; however, it was revealed that all four types showed a consensus for the following Q-item: “Q21. I should be concerned with the pandemic as it will be a relevant issue for me as a nurse (mean Z = 1.10)”. In other words, they shared a common characteristic, i.e., as nursing students in a pandemic, they were willing to study and collect educational, medical, and policy-related information for the benefit of people’s health.

## 4. Discussion

This study was conducted to categorize the emotional responses of South Korean nursing students to the pandemic and investigate the characteristics of each type of emotional response.

The study identified four types of emotional responses, “self-protection”, “pessimism about the current situation”, “realistic optimism”, and “developmental-seeking”, among South Korean nursing students.

Those who exhibited self-protection (Type 1) were willing to overcome the challenges of a pandemic; however, they demonstrated a psychologically defensive attitude. The characteristics of Type 1 affirm the findings from a previous study, which found that individuals’ perception of risks is influenced by their intuitive judgments, subjective emotions, and understanding of objective risks in the outside world [21]. In addition, the participants strongly agreed with the Q-item: “Q30. It allowed me to think about the nurses’ sense of duty”. An examination of their interviews revealed that, to them, a nurse’s sense of duty was not unconditional; rather, it implied doing one’s best to serve others while protecting oneself. Moreover, Type 1 supports the finding that an individual’s ability to perceive risks is vital for adjustment and self-protection [21]. Furthermore, having a self-protective attitude can enhance an individual’s psychological comfort and make them take precautions [22,23]. Encouraging a self-protective attitude could be part of intervention plans for the mental health wellness of healthcare providers [24,25]. According to Kim [26], a self-protective attitude can appear as a defense mechanism against stigma, and these attributes are related to individual cognitive characteristics. Hence, they are viewed as a cognitive coping method among Type 1 individuals.

A large percent (31.9%) of the participants exhibited the Type 2 response. This group expressed concerns and feelings of depression with regard to the pandemic but became indifferent as the pandemic advanced. Moreover, they demonstrated a longing for freedom, and exhibited confusion and feelings of frustration with reality. This is similar to the results of a study by Lee and Ahn [27] in which nursing students complained of feelings of isolation, embarrassment, and feeling burdened by the spread of COVID-19. Kim et al. [28] reported that nursing students in MERS situations had higher anxiety scores than adults, and that their anxiety scores were similar to those of firefighters and other frontline medical personnel. Similarly, the lockdown and social distancing measures that were implemented due to COVID-19 had a negative impact on the mental health of nursing students [29,30]. Thus, the majority of nursing students who participated in this study experienced negative emotions (when compared to the other types), which serves as evidence of the current situation and also supports the findings of previous studies. An examination of the interviews with Type 2 participants demonstrated a negative defense mechanism, which can be inferred from the following statements: “It seems like someone else’s business”, and “I am being cautious so that I do not get sick; however, there are people who get infected after throwing parties, I wish those people would just die”. The tendency to view the pandemic in an extreme manner resembling the attitude of a bystander, while also expressing feelings of concern, depression, and confusion constitutes a type of defense mechanism against negative emotional experiences. It has been reported that when college students have negative defense mechanisms, they often express emotions impulsively, judge and blame others, and suppress internal psychological conflicts or impulses [31]. This can negatively impact their ability to express psychological distress and their ability to adapt [32]. Therefore, active emotional interventions must be developed to help nursing students cope with negative emotions. Furthermore, having negative perceptions towards COVID-19 patients could act as a constraint when caring for patients with infectious diseases [33]. Therefore, it is essential to positively channel emotional issues when challenging situations arise due to a pandemic [34]. The Type 2 participants strongly agreed with the following Q-items: “Q21. I should be concerned with the pandemic as it will be a relevant issue for me as a nurse”, and “Q27. The pandemic made me realize that I must study harder to become a competent nurse”. Utilizing the findings of this research to plan an intervention could help motivate nursing students to overcome negative emotions.

The third type of emotional response (Type 3) was “realistic optimism”, which applied to 25.5% of the participants. The participants who exhibited this type of response positively perceived the unexpected spare time they gained due to the pandemic; in addition, they were proud to be nursing students. This is consistent with the findings of a previous study, which reported that nursing students were grateful for the unexpected spare time, their efforts as students of the nursing department, and the opportunity to socially interact within the department [35]. In addition, as in the research of Lee and Ahn [27], there were participants who experienced negative emotions, but could accept the reality and adapt to the changes in their daily life. With the spare time they gained due to COVID-19, nursing students were able to deeply reflect on the roles and responsibilities of a nurse (which they thought of in abstract terms during the course of their studies), as well as develop a sense of professionalism. Consequently, if such realistic optimism is adopted by students in their lives, it would help them gain emotional satisfaction, achieve self-actualization, and lead a self-directed life [36,37]. Furthermore, the development of professionalism is a significant achievement in nursing education [38]. The findings of this study can help promote the development of positive professional values in nursing students. Therefore, it would be beneficial to utilize this study to derive strategies and perform an in-depth investigation of nursing education in the current era of virtual interactions.

The fourth type of emotional response (Type 4) was “developmental-seeking”, which applied to 10.6% of the participants. The participants who exhibited this type of response perceived the pandemic to be a springboard for growth and that they must persist in equipping themselves with skills to cope better with future crises. In addition, the participants reflected on the nurse’s sense of duty, believed that overcoming the pandemic could help them experience growth as a nurse, and aimed to use the new learning methods to develop themselves further. This study’s findings regarding the Type 4 response are consistent with the results of Yang and Lee’s study [33]. The findings of Lee and Lee’s study [39] also reinforce the results of this study, where they reported that the driving force behind the post-traumatic growth of nurses after caring for COVID-19 patients was influenced by the support they received from their colleagues, family, and the public. Reflecting on present nursing care services and the care they provided to patients also helped. In this study, Type 4 participants strongly agreed with the Q-item: “Q30. It allowed me to think about the nurses’ sense of duty”. However, an examination of the interviews revealed that their idea of a nurse’s sense of duty was slightly different from that of the Type 1 participants, as their statements included phrases like, “deep awareness of the sense of duty” and “confidence in my choice of the nursing department”. From their statements, it could be inferred that the participants were influenced by the public’s appreciation of healthcare providers, and people’s reevaluation of the role of nurses [33]. In addition, the characteristic attitude that the Type 4 participants had toward the new learning methods demonstrated that nursing students are aware of the need to strengthen their technical capabilities. They are also aware that developments must be made in nursing education for the Fourth Industrial Revolution to further enhance the field of nursing [40]. Therefore, if these strengths are utilized to provide students with an in-depth education of the pandemic, and a means to acquire coping skills, nursing students will grow into competent nurses who are professionals and can easily adapt to the current situation.

Furthermore, the participants of all four types of emotional responses exhibited a common response to the following Q-item: “Q21. I should be concerned with the pandemic as it will be a relevant issue for me as a nurse”. This result is most likely based on the fact that in an unpredictable situation, people generally comprehend information better as their tendency to feel anxious increases [41]. College students, consequently, experience psychological, environmental, and behavioral changes [41,42]. Therefore, this study predicts that regardless of the type of response, it is essential to provide nursing students with accurate information and continuous as well as systematic education. This will help reduce their experiences of negative emotions and help them grow into professional nurses despite the challenges of a pandemic.

Designing and implementing intervention plans based on the results of this study will help nursing students to professionally adapt to and cope with infection-related crises that they may face as a nurse in the future. However, since the Q-methodology is concerned with individual subjectivity, it is necessary to not evaluate the derived emotional response as positive or negative. Types classified by the Q-methodology are not mutually exclusive categories, and there is a correlation between characteristics of each group. Furthermore, there is a limitation when identifying and naming the types of emotional responses since it is difficult to clearly and precisely categorize the participants’ perceptions of a phenomenon as well as their subsequent responses.

Nevertheless, this study is meaningful because it is the first study to apply the Q-methodology to categorize the emotional responses of nursing students during a pandemic. The results of this study can be applied to improve nursing education and the practical field of nursing. Further investigation of the relationships between various variables such as the types of emotional responses that nursing students have to a pandemic are required to develop a proper sense of professionalism among nursing students.

## 5. Conclusions

Through this study, four types of emotional responses to the pandemic were identified among Korean nursing students. The Q-methodology made it possible to examine academic achievement, major satisfaction, professional intuition, etc., in accordance with the emotional responses of the nursing students. Thus, this created new knowledge for improving nursing education practices.

Based on the results of this study, I would like to suggest the following. First, research is needed to explore various influencing factors on the emotional response types presented in this study. Second, it is necessary to prepare an educational strategy suitable for the current situation by reflecting on the characteristics of each type of emotional response identified in this study. Third, it is suggested that the results of this study be used for mental health education for nursing students and nurses. Fourth, it is necessary to take protective measures for nursing students or nurses who experience negative emotions or reactions, and further research is required in this area.

## Figures and Tables

**Table 1:** Eigenvalue and variance according to type.

Type	1	2	3	4
Eigenvalues	10.21	4.47	2.26	1.81
Variance (%)	8	12	13	6
Cumulative (%)	8	20	33	39

**Table 2:** Factor weight and general characteristics of P-samples according to type (*n* = 38).

Type (*n*)	ID	Gender	Age (Year)	Grade	Personality	Reason for Choosing a Department	Satisfaction with Major Selection	Factor Weight
1 (*n* = 6)	3	F	19	2	Extrovert	Ease of employment	Good	0.56
	4	F	20	2	Mixed	Voluntary	Ordinary	0.60 *
	13	M	21	2	Mixed	Ease of employment	Good	0.43
	24	F	30	3	Mixed	Voluntary	Ordinary	0.47
	33	F	18	1	Mixed	Recommendations of others	Ordinary	0.43
	46	F	20	3	Mixed	Meet college admission scores	Ordinary	0.52
2 (*n* = 15)	1	F	19	2	Mixed	Ease of employment	Good	0.68 *
	9	M	24	2	Introvert	Ease of employment	Ordinary	0.35
	15	F	20	2	Mixed	Voluntary	Ordinary	0.58
	17	F	19	2	Mixed	Voluntary	Good	0.61
	18	F	19	2	Mixed	Ease of employment	Good	0.60
	19	F	19	2	Extrovert	Ease of employment	Good	0.60
	21	F	20	3	Mixed	Ease of employment	Ordinary	0.54
	22	F	20	3	Mixed	Voluntary	Good	0.34
	26	M	25	2	Mixed	Recommendations of others	Ordinary	0.45
	28	F	19	2	Introvert	Voluntary	Good	0.57
	30	F	18	1	Extrovert	Voluntary	Good	0.52
	37	F	18	1	Extrovert	Voluntary	Ordinary	0.57
	38	F	18	1	Mixed	Voluntary	Good	0.45
	42	F	20	3	Introvert	Voluntary	Good	0.57
	45	F	21	3	Introvert	Voluntary	Ordinary	0.60
3 (*n* = 12)	5	M	21	2	Introvert	Voluntary	Ordinary	0.40
	6	F	19	2	Extrovert	Voluntary	Good	0.42
	8	M	20	2	Introvert	Voluntary	Ordinary	0.65 *
	11	M	22	2	Mixed	Ease of employment	Ordinary	0.38
	23	M	21	2	Extrovert	Voluntary	Good	0.66
	25	F	30	3	Mixed	Ease of employment	Ordinary	−0.58
	32	F	18	1	Mixed	Ease of employment	Good	0.63
	34	F	18	1	Extrovert	Voluntary	Ordinary	0.60
	36	F	19	1	Introvert	Voluntary	Good	0.53
	43	F	20	3	Introvert	Voluntary	Ordinary	0.56
	44	M	23	3	Extrovert	Recommendations of others	Ordinary	0.55
	47	F	20	3	Extrovert	Voluntary	Good	0.49
4 (*n* = 5)	10	M	22	2	Introvert	Voluntary	Good	0.46
	14	M	23	2	Mixed	Voluntary	Ordinary	−0.35
	31	F	19	1	Mixed	Voluntary	Good	0.55
	39	F	22	4	Introvert	Voluntary	Ordinary	0.60 *
	40	F	20	3	Mixed	Voluntary	Ordinary	0.38

* Typical type.

**Table 3:** Q-statements and Z-scores according to types of emotional responses.

No.	Q-Statement	Z−Scores			
		Type 1 (*n* = 6)	Type 2 (*n* = 15)	Type 3 (*n* = 12)	Type 4 (*n* = 5)
1	I am suspicious that the people I come into contact with may be infected.	0.62	0.62	−1.05	−1.47
2	I am afraid that I may spread the infection to others and be stigmatized as a spreader of an infectious disease.	1.56	−0.05	−1.02	0.30
3	I hope the pandemic will end before I become a nurse.	1.75	1.85	1.10	−0.22
4	Everyday places have changed into places of fear.	−1.11	−0.44	−0.88	0.18
5	Experiencing the pandemic made me afraid of becoming a nurse.	−0.68	−0.52	−1.21	−0.66
6	I am concerned that the quality of nursing education will decrease due to the pandemic.	−0.41	−0.71	−1.22	−1.40
7	I am depressed that my activities are being restricted due to the pandemic.	−0.45	1.44	−0.14	−0.86
8	I am afraid that I may become infected at school or clinical training sites.	−0.58	−0.09	−0.91	0.42
9	I think I am becoming weary and numb as the pandemic continues.	−0.74	0.94	−0.60	−0.52
10	I experienced physical symptoms due to the pandemic, such as loss of appetite and indigestion.	−2.36	−2.32	−1.73	−0.60
11	I am nervous about whether I will be able to cope well with the pandemic after I become a nurse.	−0.85	0.47	−0.17	−0.16
12	I am depressed because I do not know when the pandemic will end.	−0.47	0.76	−0.11	0.67
13	I feel stifled because my freedom is being suppressed.	−1.02	1.30	−1.01	0.34
14	As a nursing student, it infuriates me to see people behaving irresponsibly.	−1.16	0.58	0.44	−1.30
15	I am dissatisfied with the unilateral government and educational policies.	−0.69	−0.50	−0.19	−0.35
16	I regret enrolling in the nursing program.	0.01	−1.91	−1.95	−1.95
17	I am worried about being unprepared as a nursing student.	0.64	1.56	−0.10	0.02
18	I am afraid that I may not be able to become a nurse or get employed.	−1.09	−0.73	−1.68	0.70
19	My expectations of college life were shattered.	−1.33	0.25	0.45	−1.72
20	I am confused about whether I am doing well.	−0.75	1.10	0.48	0.96
21	I should be concerned with the pandemic as it will be a relevant issue for me as a nurse.	1.34	1.14	0.94	0.98
22	Even in a pandemic, I must do what I want to do.	0.80	−0.57	−0.71	−1.19
23	We will be able to experience personal growth by overcoming the pandemic.	0.83	0.57	0.71	1.42
24	As a nursing student, I actively educate those around me about the importance of infection control.	0.83	−0.74	0.38	0.41
25	I wish that infectious disease and disaster management courses were included in the nursing education curriculum.	0.83	0.20	0.19	0.71
26	I feel proud to be a nursing student when I see hard-working nurses.	1.53	1.23	1.40	0.70
27	The pandemic made me realize that I must study harder to become a competent nurse.	1.26	1.11	0.60	0.67
28	Due to the pandemic, I had more time to focus on myself and think about the future.	−0.47	−1.30	0.54	−0.03
29	I feel a sense of accomplishment in completing this stage of education despite difficult circumstances.	0.49	−1.00	0.77	0.02
30	It allowed me to think about the nurses’ sense of duty.	1.36	0.31	0.59	1.38
31	By pursuing nursing education, I realized the importance of infection control, and it became a way of life.	0.55	−0.12	0.99	1.10
32	As a nursing student, I feel that I should approach information objectively.	1.06	−0.56	−0.14	−0.48
33	I look forward to becoming a nurse and participating in social activities as a professional.	0.18	−0.41	2.48	1.14
34	It feels nice to have unexpected spare time.	−0.20	−1.16	1.24	−2.28
35	I think the current pandemic is a learning ground that will help us cope with future crises.	0.34	−1.02	0.91	1.16
36	It was nice to spend more time with my family due to the pandemic.	−1.09	−0.92	1.02	0.63
37	I think my competence as a professional nurse will increase as a result of experiencing new methods of teaching and learning.	−0.54	−0.37	−0.42	1.26

**Table 4:** Z-score differences by types of emotional responses.

Type	No.	Q Statements	Z-Score	Average	Difference
1	2	I am afraid that I may spread the infection to others and be stigmatized as a spreader of an infectious disease.	1.56	0.20	1.36
	32	As a nursing student, I feel that I should approach information objectively.	1.06	−0.03	1.09
	22	Even in a pandemic, I must do what I want to do.	0.80	−0.42	1.22
	16	I regret enrolling in the nursing program.	0.01	−1.45	1.46
	20	I am confused about whether I am doing well.	−0.75	0.45	1.20
2	17	I am worried about being unprepared as a nursing student.	1.56	0.53	1.03
	7	I am depressed that my activities are being restricted due to the pandemic.	1.44	0.00	1.44
	13	I feel stifled because my freedom is being suppressed.	1.30	−0.10	1.40
	9	I think I am becoming weary and numb as the pandemic continues.	0.94	−0.23	1.17
	31	By pursuing nursing education, I realized the importance of infection control, and it became a way of life.	−0.12	0.63	0.75
	24	As a nursing student, I actively educate those around me about the importance of infection control.	−0.74	0.22	0.96
	29	I feel a sense of accomplishment for completing this stage of education despite difficult circumstances.	−1.00	0.07	1.07
	35	I think the current pandemic is a learning ground that will help us cope with future crises.	−1.02	0.35	1.37
	34	It feels nice to have unexpected spare time.	−1.16	−0.60	0.56
	28	Due to the pandemic, I had more time to focus on myself and think about the future.	−1.30	−0.32	0.98
3	33	I look forward to becoming a nurse and participating in social activities as a professional.	2.48	0.85	1.63
	34	It feels nice to have unexpected spare time.	1.24	−0.60	1.84
	2	I am afraid that I may become a spreader of an infectious disease and be stigmatized as one.	−1.02	0.20	1.22
4	37	I think my competence as a professional nurse will increase as a result of experiencing new methods of teaching and learning.	1.26	−0.02	1.28
	3	I hope the pandemic will end before I become a nurse.	−0.22	1.12	1.34
	10	I experienced physical symptoms due to the pandemic, such as loss of appetite and indigestion.	−0.60	−1.75	1.15
	34	It feels nice to have unexpected spare time.	−2.28	−0.60	1.68

## Data Availability

The collected data were sealed and stored on a password-protected hard drive that could only be accessed by the principal investigator. It was decided that this data would be retained for three years, from the time that the data were collected until the publication of the thesis. The data will be destroyed after the publication.
